# Malignant changes developing from odontogenic cysts: A systematic review

**DOI:** 10.4317/jced.53256

**Published:** 2016-12-01

**Authors:** Jordi Borrás-Ferreres, Alba Sánchez-Torres, Cosme Gay-Escoda

**Affiliations:** 1 DDS. Fellow of the Master’s Degree Program in Oral Surgery (EHFRE International University/FUCSO); 2 DDS. Fellow of the Master of Oral Surgery and Orofacial Implantology. School of Dentistry, University of Barcelona, Spain; 3 MD, DDS, MS, PhD, EBOS. Chairman and Professor of the Oral and Maxillofacial Surgery Department, School of Dentistry, University of Barcelona. Director of Master’s Degree Program in Oral Surgery and Implantology (EHFRE International University/FUCSO). Coordinator/Researcher of the IDIBELL Institute. Head of Oral and Maxillofacial Surgery and Implantology Department of the Teknon Medical Centre, Barcelona, Spain

## Abstract

The aim of this study was to systematically review scientific literature in orderto describe the characteristics and prognosis of malignant entities developing from odontogenic cysts. A search in Pubmed (MEDLINE) and Cochrane databases was conducted. The inclusion criteria were articles published in English related to the malignisation of odontogenic cysts in humans. The exclusion criteria were articles that do not specify the type of odontogenic cyst, malignisation of parakeratinised keratocysts, the presence of an ameloblastic carcinoma and metastasis from distant primary tumours. The selected articles were classified according to Strength of Recommendation Taxonomy criteria. Statistical analysis of the data was carried out using statistical package software SPSS version 22.0. From the 1,237 articles initially obtained, the authors included 3 case series and 45 case reports in the end. Descriptive analysis showed that men have a disposition for malignisation from odontogenic cysts and they frequently appear at the posterior mandible, with pain and swelling being the most frequent signs and symptoms. Follicular cysts were the entities that underwent the most malignant changes with well differentiated squamous cell carcinomas being the most prevalent type of malignancy. The real prognosis of this malignancy is not known because of the heterogeneity of available studies.

** Key words:**Odontogenic cysts, squamous cell carcinoma, neoplastic cell transformation, oral cancer.

## Introduction

Odontogenic cysts are the most frequent lesions appearing in the jaws. They are defined as cavities filled with liquid, semiliquid or gaseous content with odontogenic epithelial lining and connective tissue on the outside (1). They originate from the epithelial component of the odontogenic apparatus or its remnants that lie entrapped within the bone or in the peripheral gingival tissues (2). Although odontogenic cysts are benign lesions, carcinomatous degeneration has been described in the literature with an incidence that ranges from 0.13% to 3% (3-6). The different types of carcinomatous changes that may develop from odontogenic cysts have been widely grouped as subtypes of primary intraosseous carcinomas (PIOC), uncommon jaw malignancies derived from odontogenic epithelial remnants (5,7). The most common symptoms in these malignant tumours are pain and swelling, although in some cases the patient can be asymptomatic with the lesion being found through a routine dental panoramic radiography (8). Unfortunately, the absence of symptoms leads to a delay in clinical diagnosis thereby hindering oral cancer prognosis (9). Although malignisation does not frequently appear, clinicians must know the main factors related to these lesions.

The aim of this study was to perform a systematic review of the different malignant entities developing directly from odontogenic cysts reported in scientific literature and to describe their characteristics and prognosis. In order to find the appropriate literature, the following PICO question was formulated: “Among patients who have suffered from a malignant change from a pre-existing odontogenic cyst, what are the characteristics of the neoplasm and its prognosis?”

## Material and Methods

This study is a systematic review of the literature as a whole with regard to the malignisation of odontogenic cysts. This article follows the Preferred Reporting Items for Systematic Reviews and MetaAnalyses (PRISMA) declaration (10).

An electronic search in Pubmed (MEDLINE) and the Cochrane Library was conducted between January 2015 and April 2015. The inclusion criteria were: a) articles related to the malignisation of odontogenic cysts in humans and b) publications in English. The exclusion criteria were: a) articles not conforming to the type of odontogenic cyst, b) malignisation of parakeratinised keratocysts, as they have recently been renamed keratocystic odontogenic tumours and considered to be benign since the new WHO classification was created in 2005 (11), c) the presence of an ameloblastic carcinoma and d) carcinomatous changes belonging to metastasis from distant primary tumours.

The search strategy was “Odontogenic Cysts”[Mesh] OR “Jaw Cysts”[Mesh] OR “Bone Cysts”[Mesh] OR “Dentigerous Cyst”[Mesh] OR “Radicular Cyst”[Mesh] OR residual cyst AND “Carcinoma, Squamous Cell”[Mesh] OR “Carcinoma, Mucoepidermoid”[Mesh] NOT keratocystic odontogenic tumour NOT Gorlin Syndrome.

Articles were selected independently by two of the authors and the results were then compared. If differences were noted, the authors reached an agreement. The selected articles were classified into different levels of evidence by means of the Strength of Recommendation Taxonomy (SORT) criteria (12).

The characteristics collected from the studies to do the quantitative analysis were based on: age, gender, signs and symptoms, radiologic assessment, location, presumed odontogenic cyst type, histopathological results, treatment and patient status.

A descriptive analysis was performed using statistical package SPSS 22.0 software (IBM Corp, Armonk, NY).

## Results

**Figure 1:**
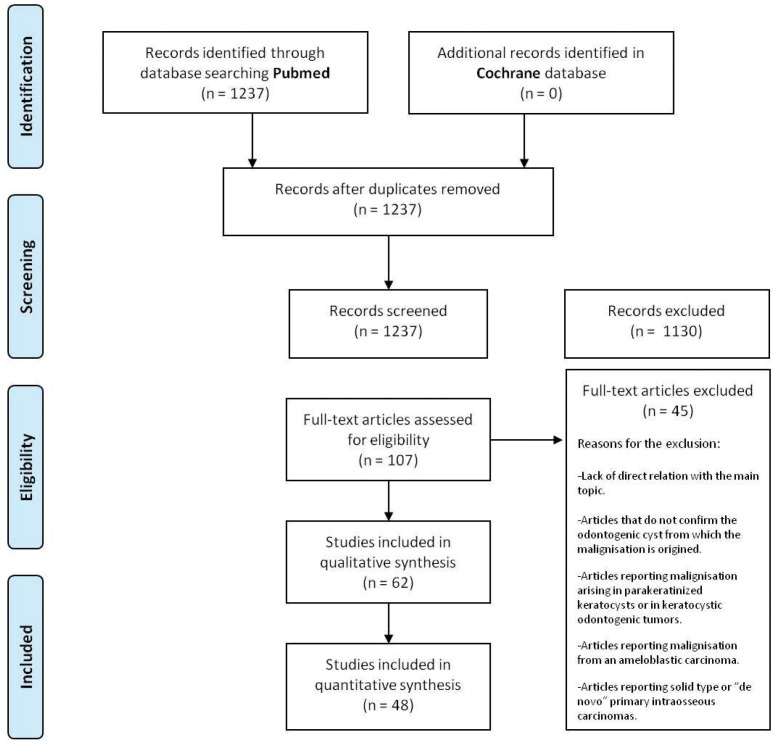
Flow of articles through the systematic review according to PRISMA statement.

The authors obtained 1,237 articles from the initial search. One hundred and seven articles were chosen for complete text analysis by screening titles and abstracts. However, the full text of 20 articles could not be obtained so they were excluded. Finally, 48 relevant articles were selected to be included in the systematic review: 3 case series (13-15) and 45 case reports (4-8,13,15-59).Despite the fact that all these studies had a scientific level 3 and no randomised clinical trials could be found, the authors decided to include them in order to analyse the available literature. The flow chart of the selected articles and the main reasons for their exclusion can be seen on figure 1. The main characteristics of the included studies are shown on  Table 1.

**Table 1:**
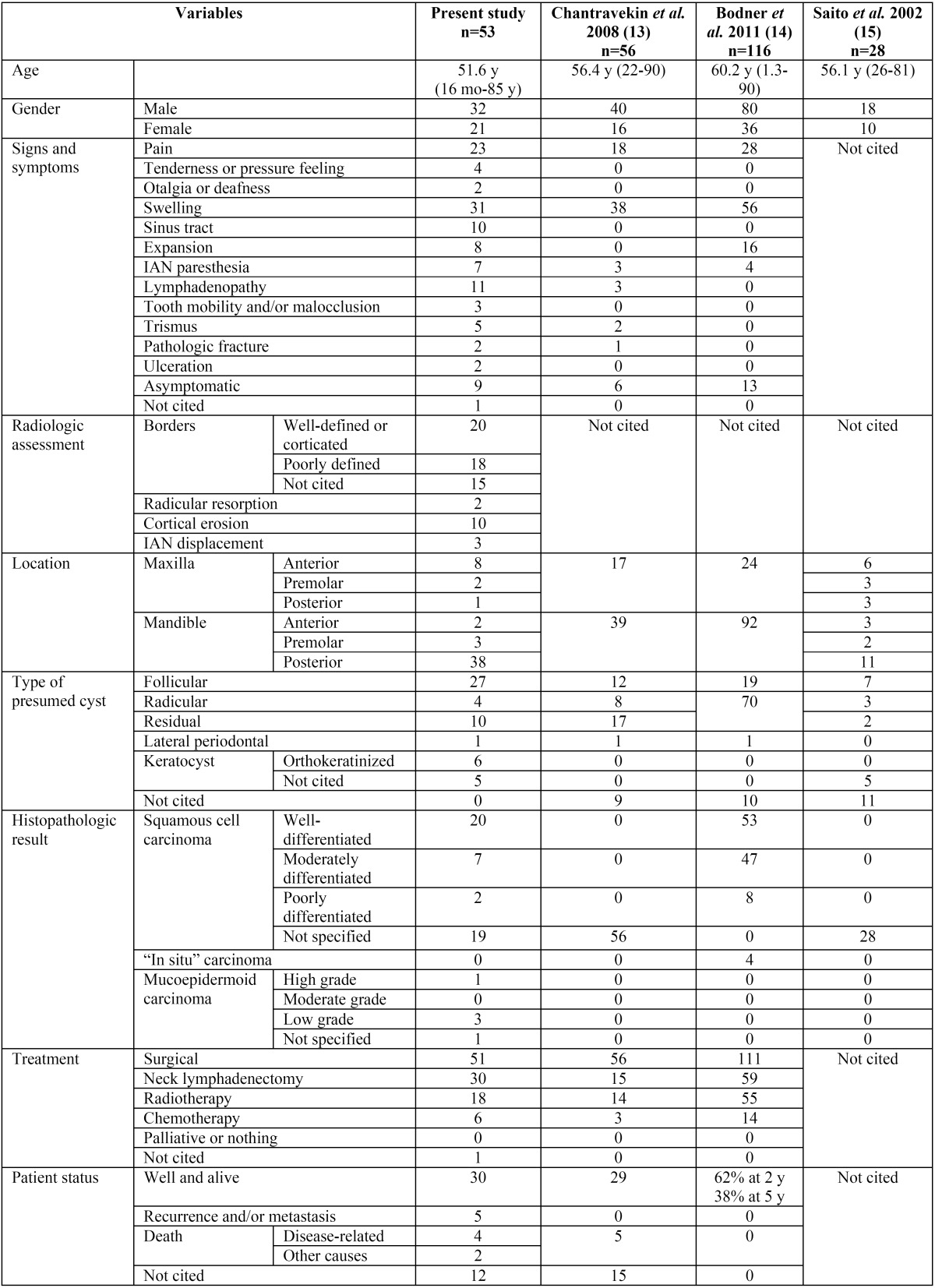
Characteristics of the included studies. Mo: Months, w: weeks, y: years, IAN: inferior alveolar nerve.

Concretely, 53 isolated cases were found and grouped as a case series to then be compared with the other series found. It should be noted that the work carried out by Chantravekin *et al.* (13) included 9 cases of keratocystic odontogenic tumours and the article from Bodner *et al.* (14) included 16 keratocystic odontogenic tumours, 3 verrucous carcinoma and 1 spindle cell carcinoma. None of them could be excluded from all the categories shown on  Table 1 because of the lack of detailed and individual data in these studies.

From the 53 cases retrieved, a predominance for malignancy was found in men (man:woman ratio = 1.52:1). The mean age was 51.6 years (range: 16 months – 85 years). No differences between races were observed. The main symptoms related to the pathology were swelling (n=31, 58.5%) and pain (n=23, 43.4%). However, 9 cases (17%) were asymptomatic. Radiologically, minimal differences could be observed with regard to the corticated (n=20, 37.7%) or diffused (n=18, 33.9%) borders, although 15 cases were not described. Cortical erosion (n=10, 18.8%) occurred frequently. The posterior mandible was the most affected site (n=38, 71.7%). With regard to cyst type, follicular (n=27, 50.9%) and residual cysts (n=10, 18.8%) were the most common and the well differentiated squamous cell carcinoma (n=20, 37.7%) was the most prevalent malignancy. A good number of patients were treated with a surgical excision of the lesion (96.2%) and more than 50% of them required a neck lymphadenectomy (56.6%).

More than 70% of patients were alive (n=30, 73.2%) in a time-period ranging from 4 months to 10 years after follow-up, 5 (9.4%) had recurrences and/or metastasis in a period of time ranging from 2 to 6 years (there were 2 cases without follow-up) and 4 (7.5%) had a disease-related death between 5 months and 1 year. Unfortunately, the status of 12 patients was not cited.

Six patients (11.3%) developed a carcinomatous degeneration after a previous cystectomy with or without tooth extraction.

**Figure 2:**
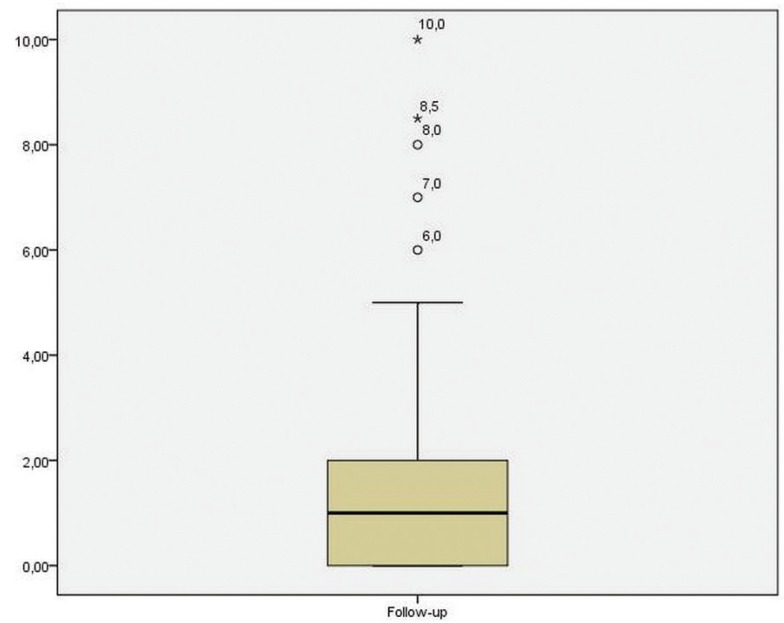
Box diagram that shows the time of follow-up (years) reported by the included case reports. Mean 1.8 years (CI95% [1.16 to 2.45]).

As shown in figure 2, the average follow-up time was 1.8 years (CI95% [1.16 to 2.45]) ranging between 3 weeks and 10 years. However, 14 case reports did not specify the follow-up.

## Discussion

Odontogenic carcinomas are extremely rare tumours that develop from remnants of odontogenic epithelium. At the time of diagnosis, they specifically affect the bone and it is mandatory to discard any infiltration to the surrounding tissues and the likelihood of a distant metastasis originating from a primary tumour (59). In the last WHO classification made in 2005, the term “primary intraosseous squamous cell carcinoma” was formulated (PIOSCC) which include classic types 1 and 3 primary intraosseous carcinomas, thus leaving malign ameloblastoma, ameloblastic carcinoma and central mucoepidermoid carcinoma as separate conditions (11). Finally, the PIOSCC was sub-classified into three types 1) ex-cystic PIOSCC, 2) PIOSCC deriving from keratocystic odontogenic tumours and 3) solid or “de novo” PIOSCC (55). The diagnostic criteria for type 1 PIOSCC, proposed by Gardner (60) in 1975, includes the observation of the transition between the cystic epithelial lining and the invasive squamous cell carcinoma. However, the transition between the cystic lining and the carcinoma is more likely to be observed at the first stages of malign transformation. Its identification in more advanced stages can be difficult (18) and there are even some cases of type 1 PIOSCC being diagnosed although the transition was not observed (19,41).

The incidence of carcinomas, either squamous or mucoepidermoid, originating from odontogenic cysts represents less than 1% (5,19). According to Muller and Waldron (34), 70% of primary intraosseous carcinomas develop from pre-existing cysts and these account for 1 to 2% of overall oral cancers (15,42,43). However, it has been reported that up to 50% of CMEC originates from odontogenic cysts or impacted teeth (61).

The pathogenesis is still not known although the presence of infectious tissue has been related to it (4). Long periods of chronic inflammation have been suggested to be a predisposing factor to the malign transformation of the cystic epithelium (5,18,45-48). However, Gulbranson *et al.* (49) published a case report of malignisation from a follicular cyst without chronic inflammation in a very young woman which could indicate the existence of an additional physiopathological mechanism related to the oncogenes (5,49). Two of the included case series (13,14) found that the majority of cases had originated from radicular or residual cysts (both inflammatory cysts). To the contrary, we have found higher prevalence of malignant changes developing from follicular cysts (50.9%), followed by residual (18.8%) and radicular cysts (7.5%). Browne *et al.* (62) and van der Wal *et al.* (50) suggested that the existence of keratinisation in the epithelial lining could be a risk factor for malignancy. Although keratinisation in odontogenic cysts has only been observed in 15 to 18% of cases (16), the majority of type 1 PIOSCC are keratinised and well-differentiated. Accordingly, a great proportion (37.7%) of well-differentiated carcinomas was found in our study.

A former case series (14) described 116 cases of type 1 PIOSCC. The average age was 60.2 years (1.3 to 90 years) and the man: woman ratio was 2.22:1. The authors state that men’s disposition is not specific to type 1 PIOSCC, but it can be explained by the greater incidence of odontogenic cysts in men. Like the case series included (13-15), we have found more cases in men than in women (30 versus 18, respectively) and the mean age of apparition of these malignancies tends to be between the 5th and 6th decade of life. Conversely, scientific literature describes CMEC to be twice as frequent in females with an age range that varies from 1 to 78 years and a higher incidence in the 4th and 5th decade (51). However, we only have five cases of ex-cyst CMEC, 3 in women and 2 in men. Thus, this reduced sample does not allow us to draw any conclusions. No comparisons can be made since no cases with the development of this malignant entity were found in the case series (13-15).

The most frequently reported signs and symptoms for type 1 PIOSCC are pain and swelling (5,7,8,14,34,42,43,47-49) as found in our study, followed by dental mobility (51), cortical perforation (5,14), adherence of the cystic lining to the bone cavity (14,16), the delay of alveolar healing after a tooth extraction (17,47) and even the presence of chronic sinus tracts (5,14). Lymphadenopathy and sensorial perturbances such as paresthesia or numbness have been reported less frequently (5,8) although in this study we found 20.7% lymphadenopathy, a percentage similar to chronic sinus tracts (18.8%) and cortical perforation (18.8%). It is noteworthy that 17% of the isolated cases included were asymptomatic.

Radiographically, the malignant change of an odontogenic cyst may not be well distinguished in the early stages, though it must be considered when fast growth of an area occurs (47). It usually tends to appear as a unilocular radiolucency with irregular sca-lloped and poorly defined edges suggesting invasive behaviour, especially if the osseous cortical is eroded (4-8,13,16,19,34,47-49,51). However, this study has found similar percentages of corticated (37.7%) and poorly defined (33.9%) edges. Unfortunately, the other case series (13-15) included did not specify the radiologic pattern. Although orthopantomography is an essential diagnostic tool, it has some limitations for diagnosing some lesions because of the superposition of images or the lack of information on soft tissues. Sometimes small asymptomatic but malign lesions can be misdiagnosed. On the other hand, computed tomography shows the real extension, in other words, the invasiveness to soft tissues, cortical destruction and edge type (19). Since computed tomography is not routinely used, some malignant lesions are diagnosed after their elimination and an anatomopathological exam (53).

The delay in healing after a cystectomy with or without dental extraction could indicate malignancy (47,48). However, some malignant cases with the complete healing of the soft tissues have been described. Thus, histological analysis of the whole specimen must be done (4,18,43,48). The delay in diagnosis negatively influences the prognosis (42,51). Interestingly, we have found 6 cases of previous dental extractions which later developed malignant changes. The most frequent associated symptoms were swelling (n=3, 50%), pain (n=2, 33.3%) and the presence of a sinus tract (n=2, 33.3%), inaccordance with the symptoms related to the overall cases. Only 1 case was asymptomatic.

The results found in this study suggest that the majority of patients (73.1%) are still alive after a period of time between 4 months and 10 years. Likewise, the case series published by Chantravekin *et al.* (13) and Bodner *et al.* (14) had higher percentages of patient survival, 85.3% and 62% at 2 years, respectively. Nevertheless, these studies did not report recurrence/metastasis cases while we found 5 cases.

Finally, some of the limitations of this study need to be discussed. First, the articles included were all case reports and case series, all with scientific level 3. In addition, it is worth mentioning that the case series (13-15) found did not register all the variables collected in this study. Some of them (13,14) showed cases of malignancies that had not developed from odontogenic cysts and could not be excluded due to the lack of detailed information in the entire text. The real prognosis of these entities is difficult to know due to different follow-up times carried out in the included studies. The majority of studies control their cases for up to 2 years and this may include some recurrence or metastases cases developed after more time.

The fact that these types of lesions rarely appear determines the importance of awareness and knowing their characteristics. With regard to the implications for research, there is a need for clinical studies in larger populations in order to significantly expand current knowledge on malignant changes developing from odontogenic cysts.

## Conclusions

- Men have a predisposition for malignant changes in odontogenic cysts that frequently appear at the posterior mandible. The most frequent signs and symptoms are pain and swelling, although some cases can be asymptomatic.

- The real prognosis of these malignancies is not known due to the heterogeneity of the included studies with regard to different follow-up periods and even some studies did not report it.

## References

[1] Nuñez-Urrutia S, Figueiredo R, Gay-Escoda C (2010). Retrospective clinicopathological study of 418 odontogenic cysts. Med Oral Patol Oral Cir Bucal.

[2] Mosqueda-Taylor A, Irigoyen-Camacho ME, Diaz-Franco MA, Torres-Tejero MA (2002). Odontogenic cysts. Analysis of 856 cases. Med Oral.

[3] Sharifian MJ, Khalili M (2011). Odontogenic cysts: a retrospective study of 1227 cases in an Iranian population from 1987 to 2007. J Oral Sci.

[4] Scheer M, Koch AM, Drebber U, Kübler AC (2004). Primary intraosseous carcinoma of the jaws arising from an odontogenic cyst--a case report. J Craniomaxillofac Surg.

[5] Jain M, Mittal S, Gupta DK (2013). Primary intraosseous squamous cell carcinoma arising in odontogenic cysts: an insight in pathogenesis. J Oral Maxillofac Surg.

[6] Swinson BD, Jerjes W, Thomas GJ (2005). Squamous cell carcinoma arising in a residual odontogenic cyst: case report. J Oral Maxillofac Surg.

[7] Charles M, Barr T, Leong I, Ngan BY, Forte V, Sándor GK (2008). Primary intraosseous malignancy originating in an odontogenic cyst in a young child. J Oral Maxillofac Surg.

[8] Chaisuparat R, Coletti D, Kolokythas A, Ord RA, Nikitakis NG (2006). Primary intraosseous odontogenic carcinoma arising in an odontogenic cyst or de novo: a clinicopathologic study of six new cases. Oral Surg Oral Med Oral Pathol Oral Radiol Endod.

[9] Levi PA Jr, Kim DM, Harsfield SL, Jacobson ER (2005). Squamous cell carcinoma presenting as an endodontic-periodontic lesion. J Periodontol.

[10] Moher D, Liberati A, Tetzlaff J, Altman DG, The PRISMA Group (2009). Preferred Reporting Items for Systematic Reviews and Meta-Analyses: The PRISMA Statement. PLoS Med.

[11] Eversole LR, Siar CH, van derWaal I (2005). Primary intraosseous squamous cell carcinomas. In: Barnes L, Evson JW, Reichart P, Sidransky D. World Health Organization classification of tumors. Pathology and genetics head and neck tumors. World Health Organization International Agency for Research on Cancer. Lyon: IACR Press.

[12] Ebell MH, Siwek J, Weiss BD, Woolf SH, Susman J, Ewigman B (2004). Strength of recommendation taxonomy (SORT): A patient-centered approach to grading evidence in the medical literature. J Am Board Fam Pract.

[13] Chantravekin Y, Rungsiyanont S, Tang P, Tungpisityotin M, Swasdison S (2008). Primary intraosseous squamous cell carcinoma derived from odontogenic cyst: Case report and review of 56 cases. Asian J Oral Maxillofac Surg.

[14] Bodner L, Manor E, Shear M, van der Waal I (2011). Primary intraosseous squamous cell carcinoma arising in an odontogenic cyst: a clinicopathologic analysis of 116 reported cases. J Oral Pathol Med.

[15] Saito T, Okada H, Akimoto Y, Yamamoto H (2002). Primary intraosseous carcinoma arising from an odontogenic cyst: a case report and review of the Japanese cases. J Oral Sci.

[16] Yoshida H, Onizawa K, Yusa H (1996). Squamous cell carcinoma arising in association with an orthokeratinized odontogenic keratocyst. Report of a case. J Oral Maxillofac Surg.

[17] McDonald AR, Pogrel MA, Carson J, Regezi J (1996). p53-positive squamous cell carcinoma originating from an odontogenic cyst. J Oral Maxillofac Surg.

[18] Yasuoka T, Yonemoto K, Kato Y, Tatematsu N (2000). Squamous cell carcinoma arising in a dentigerous cyst. J Oral Maxillofac Surg.

[19] Cavalcanti MG, Veltrini VC, Ruprecht A, Vincent SD, Robinson RA (2005). Squamous-cell carcinoma arising from an odontogenic cyst--the importance of computed tomography in the diagnosis of malignancy. Oral Surg Oral Med Oral Pathol Oral RadiolEndod.

[20] Baker RD, D'Onofrio ED, Corio RL, Crawford BE, Terry BC (1979). Squamous-cell carcinoma arising in a lateral periodontal cyst. Oral Surg Oral Med Oral Pathol.

[21] Bradley N, Thomas DM, Antoniades K, Anavi Y (1988). Squamous cell carcinoma arising in an odontogenic cyst. Int J Oral Maxillofac Surg.

[22] Cox DP (2012). p53 expression and mutation analysis of odontogenic cysts with and without dysplasia. Oral Surg Oral Med Oral Pathol Oral Radiol.

[23] Dabbs DJ, Schweitzer RJ, Schweitzer LE, Mantz F (1994). Squamous cell carcinoma arising in recurrent odontogenic keratocyst: case report and literature review. Head Neck.

[24] Epstein JB, Hollender L, Pruzan SR (2004). Mucoepidermoid carcinoma in a young adult: recognition, diagnosis, and treatment and responsibility. Gen Dent.

[25] High AS, Quirke P, Hume WJ (1987). DNA-ploidy studies in a keratocyst undergoing subsequent malignant transformation. J Oral Pathol.

[26] Johnson LM, Sapp JP, McIntire DN (1994). Squamous cell carcinoma arising in a dentigerous cyst. J Oral Maxillofac Surg.

[27] Lapin R, Garfinkel AV, Catania AF, Kane AA (1973). Squamous cell carcinoma arising in a dentigerous cyst. J Oral Surg.

[28] Martinelli C, Melhado RM, Callestini EA (1977). Squamous-cell carcinoma in a residual mandibular cyst. Oral Surg Oral Med Oral Pathol.

[29] Minić AJ (1992). Primary intraosseous squamous cell carcinoma arising in a mandibular keratocyst. Int J Oral Maxillofac Surg.

[30] Nithiananda S (1983). Squamous cell carcinoma arising in the lining of an odontogenic cyst. Br J Oral Surg.

[31] Siar CH, Ng KH (1987). Squamous cell carcinoma in an orthokeratinised odontogenic keratocyst. Int J Oral Maxillofac Surg.

[32] Van der Waal I, Rauhamaa R, Van der Kwast WA, Snow GB (1985). Squamous cell carcinoma arising in the lining of odontogenic cysts. Report of 5 cases. Int J Oral Surg.

[33] Spoorthi BR, Rao RS, Rajashekaraiah PB, Patil S, Venktesaiah SS, Purushothama P (2013). Predominantly cystic central mucoepidermoid carcinoma developing from a previously diagnosed dentigerous cyst: case report and review of the literature. Clin Pract.

[34] Araújo JP, Kowalski LP, Rodrigues ML, de Almeida OP, Lopes Pinto CA, Alves FA (2014). Malignant transformation of an odontogenic cyst in a period of 10 years. Case Rep Dent.

[35] Muglali M, Sumer AP (2008). Squamous cell carcinoma arising in a residual cyst: a case report. J Contemp Dent Pract.

[36] Zapała-Pośpiech A, Wyszyńska-Pawelec G, Adamek D, Tomaszewska R, Zaleska M, Zapała J (2013). Malignant transformation in the course of a dentigerous cyst: a problem for a clinician and a pathologist. Considerations based on a case report. Pol J Pathol.

[37] Roofe SB, Boyd EM Jr, Houston GD, Edgin WA (1999). Squamous cell carcinoma arising in the epithelial lining of a dentigerous cyst. South Med J.

[38] Ota Y, Karakida K, Watanabe D, Miyasaka M, Tsukinoki K (1998). A case of central carcinoma of the mandible arising from a recurrent odontogenic keratocyst: delineation of surgical margins and reconstruction with bilateral rectus abdominis myocutaneous free flaps. Tokai J ExpClin Med.

[39] Lavery K, Blomquist JE, Awty MD, Stevens PJ (1987). Squamous carcinoma arising in a dental cyst. Br Dent J.

[40] Areen RG, McClatchey KD, Baker HL (1981). Squamous cell carcinoma developing in an odontogenic keratocyst. Report of a case. Arch Otolaryngol.

[41] Nomura T, Monobe H, Tamaruya N, Kishishita S, Saito K, Miyamoto R (2013). Primary intraosseous squamous cell carcinoma of the jaw: Two new cases and review of the literature. Eur Arch Otorhinolaryngol.

[42] Rius J, Bosch JM, Uribarri A, Berini L, Gay-Escoda C (1995). Carcinoma intraóseo primario del maxilar superior originado en un quiste folicular: presentación de un caso y revisión de la literatura. Rev Act Odontoestomatol Esp.

[43] Murillo-Cortes J, Etayo-Perez A, Sebastian-Lopez C, Martino-Gorbea R, Rodriguez-Cortel JM (2002). Primary intraosseous carcinoma arising in a mandibular cyst. Med Oral.

[44] Aggarwal P, Saxena S (2011). Aggressive growth and neoplastic potential of dentigerous cysts with particular reference to central mucoepidermoid carcinoma. Br J Oral Maxillofac Surg.

[45] Foley WL, Terry BC, Jacoway JR (1991). Malignant transformation of an odontogenic keratocyst: report of a case. J Oral Maxillofac Surg.

[46] Maxymiw WG, Wood RE (1991). Carcinoma arising in a dentigerous cyst: a case report and review of the literature. J Oral Maxillofac Surg.

[47] Manganaro AM, Cross SE, Startzell JM (1997). Carcinoma arising in a dentigerous cyst with neck metastasis. Head Neck.

[48] Torrades-Ferrer M, Gay-Escoda C (1992). Carcinoma primario intraósea de mandíbula con origen en un quiste odontogénico. Rev Act Odontoestomatol Esp.

[49] Gulbranson SH, Wolfrey JD, Raines JM, McNally BP (2002). Squamous cell carcinoma arising in a dentigerous cyst in a 16-month-old girl. Otolaryngol Head Neck Surg.

[50] van der Wal KG, de Visscher JG, Eggink HF (1993). Squamous cell carcinoma arising in a residual cyst. A case report. Int J Oral Maxillofac Surg.

[51] Aboul-hosn Centenero S, Marí-Roig A, Piulachs-Clapera P, Juárez-Escalona I, Monner-Diéguez A, Díaz-Carandell A (2006). Primary intraosseous carcinoma and odontogenic cyst. Three new cases and review of the literature. Med Oral Patol Oral Cir Bucal.

[52] Holsinger FC, Owens JM, Raymond AK, Myers JN (2002). Central mucoepidermoid carcinoma of the mandible: tumorigenesis within a keratocyst. Arch Otolaryngol Head Neck Surg.

[53] Colbert S, Brennan PA, Theaker J, Evans B (2012). Squamous cell carcinoma arising in dentigerous cysts. J Craniomaxillofac Surg.

[54] Darling MR, Wehrli BM, Ciavarro C, Daley TD (2008). Pericoronal radiolucency in the posterior mandible. Oral Surg Oral Med Oral Pathol Oral Radiol Endod.

[55] Sciubba JJ, Eversole LR, Slootweg PJ (2005). Odontogenic/ameloblastic carcinomas. In: Barnes L, Eveson J, Reichart P, Sidransky D. World Health Organization classification of tumours, pathology and genetics: head and neck tumors.

[56] Yoon HJ, Hong SP, Lee JI, Lee SS, Hong SD (2009). Ameloblastic carcinoma: An analysis of 6 cases with review of the literature. Oral Surg Oral Med Oral Pathol Oral Radiol Endod.

[57] Tapia JL, Aguirre A, Garvey M, Zeid M (2004). Mandibular unilocular radiolucency with ill-defined borders. Oral Surg Oral Med Oral Pathol Oral Radiol Endod.

[58] Hayashido Y, Yoshioka Y, Shintani T, Hamana T, Koizumi K, Ishida Y (2005). Primary intraosseous carcinoma of mandible associated with elevation of serum carcinoembryonic antigen level. Oral Oncol.

[59] Waldron CA, Mustoe TA (1989). Primary intraosseous carcinoma of mandible with probable origin in an odontogenic cyst. Oral Surg Oral Med Oral Pathol.

[60] Gardner AF (1975). A survey of odontogenic cyst and their relationship to squamous cell carcinoma. J Can Dent Assoc.

[61] Eversole LR, Sabes WR, Rovin S (1975). Aggressive growth and neoplastic potential of odontogenic cysts: with special reference to central epidermoid and mucoepidermoid carcinomas. Cancer.

[62] Browne RM, Gough NG (1972). Malignant change in the epithelium lining odontogenic cysts. Cancer.

